# A two-fluid model for the macroscopic behavior of polar nematic fluids and gels in a nonchiral or a chiral solvent

**DOI:** 10.1140/epje/s10189-022-00172-8

**Published:** 2022-02-26

**Authors:** Helmut R. Brand, Harald Pleiner

**Affiliations:** 1 Department of Physics, University of Bayreuth, 95440 Bayreuth, Germany; 2 Max Planck Institute for Polymer Research, 55021 Mainz, Germany

## Abstract

**Abstract:**

We present the macroscopic dynamics of polar nematic liquid crystals in a two-fluid context. We investigate the case of a nonchiral as well as of a chiral solvent. In addition, we analyze how the incorporation of a strain field for polar nematic gels and elastomers in a solvent modifies the macroscopic dynamics. It turns out that the relative velocity between the polar subsystem and the solvent gives rise to a number of cross-coupling terms, reversible as well as irreversible, unknown from the other two-fluid systems considered so far. Possible experiments to study those novel dynamic cross-coupling terms are suggested. As examples we just mention that gradients of the relative velocity lead, in polar nematics to heat currents and in polar cholesterics to temporal changes of the polarization. In polar cholesterics, shear flows give rise to a temporal variation in the velocity difference perpendicular to the shear plane, and in polar nematic gels uniaxial stresses or strains generate temporal variations of the velocity difference.

**Graphical abstract:**

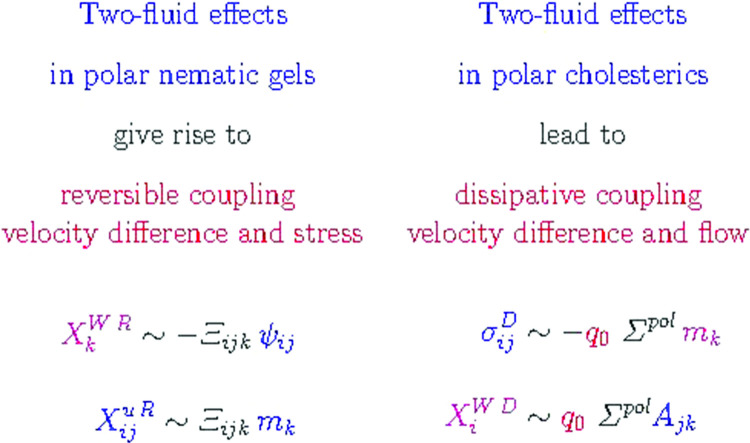

## Introduction

There are many two-fluid systems composed of two immiscible subsystems including, as examples, fluid emulsions [1], colloidal suspensions [2], polymer solutions and mixtures [3], fiber networks in a matrix [4, 5], polymeric materials reinforced by carbon nanotubes [6], and microtubules coupled to the cytoskeleton in cells [7]. Over the years, a large amount of work went into the optimization of the static properties of such materials.

More recently, macroscopic dynamic two-fluid descriptions have been given for a number of soft matter materials and complex fluids starting with immiscible liquids [8, 9] and combinations of ordinary or viscoelastic liquids with nematic liquid crystals [8]. More recently, this approach has been applied to a number of other two-fluid systems including immiscible compound materials in solids and gels [10], bioinspired complex fluids [11, 12], and materials characterized by the formation of clusters, for example of smectic clusters above the nematic to smectic *A* transition [13] and of clusters above the glass transition [14].

In macroscopic dynamics one keeps, in addition to the locally conserved variables (e.g., mass density, density of momentum and energy density) and the variables associated with spontaneously broken continuous symmetries [15–18], also macroscopic variables that relax on a sufficiently long, but finite time scale to be of interest for the macroscopic behavior of the system [18]. In the present paper, we focus predominantly on two-fluid systems with a static polar preferred direction, which breaks parity symmetry, but is invariant under time reversal. Such systems are characterized by a polar unit vector, ${\hat{p}}_i$ with ${\hat{p}}_i^2 = 1$, which breaks spontaneously rotational symmetry. It is associated with a macroscopic electric polarization $P_i = P {\hat{p}}_i$ with the magnitude *P* of the polarization. The latter is treated as a macroscopic, slowly relaxing variable. For a discussion of the molecular foundations of polar nematic order and their physical consequences, cf. [19–21].

In the case of two-fluid systems, one takes into account a second velocity, or conveniently the difference, $w_i$, of the velocities of the two subsystems. In a nonsuperfluid system, only the barycentric velocity is truly hydrodynamic and $w_i$ a macroscopic, slowly relaxing variable. In superfluids, not considered here the superfluid velocity is associated with broken gauge invariance and therefore a truly hydrodynamic quantity [22], giving rise, for example, to the propagation of second sound in the bulk in the long wavelength limit in the superfluid phases of $^4$He [22, 23] and of $^3$He [24–28].

In addition, there is a second mass density associated with the solvent system, which we will treat as a conserved quantity, although generally, depending on the material of interest, it could relax on a finite time scale [8].

In the first part of this paper, we deal with the two-fluid description of polar nematic fluids discussing two cases separately. First we consider a nonchiral solvent in Sect. 2; in this case, the corresponding one-fluid system is a polar nematic, whose macroscopic dynamics has been derived a number of years ago [29]. In Sect. 3 we investigate a two fluid model for polar nematics in a chiral solvent. These results also apply to a polar cholesteric in a nonchiral solvent, since the two fluid systems are assumed to be mixed on a microscopic scale.

In Sect. 4, we analyze how for polar nematic gels and elastomers the elastic strain field can be incorporated in a two-fluid description involving a nonchiral as well as achiral solvent. In addition, relative rotations of the polar direction with respect to the elastic network are considered. In Sect. 5, we discuss some possible dynamic experiments involving the relative velocity. In particular, reversible effects connected with heat and concentration currents in polar nematics and polar cholesterics, dissipative effects coupling to the stress tensor in polar cholesterics, and reversible coupling effects to strains and relative rotations in polar gels. A brief summary and perspective in Sect. 6 concludes the paper.

Throughout the paper we comment which static and dynamic cross-coupling terms survive the transition from polar to nonpolar nematics or cholesterics.

## Two-fluid model for polar nematics with a nonchiral solvent

### Variables

In this section of the manuscript, we study a nonchiral two-fluid system, namely a polar nematic with an isotropic liquid as the second, “solvent” fluid. For the polar nematic aspects, we will make use of the previous work on the macroscopic description of one-component polar nematics [29]. The two-fluid character is manifest by the additional macroscopic variables, velocity difference $w_i$ and concentration $\phi$ of the solvent component [8, 9]. The other variables are the entropy density, $\sigma$, the density, $\rho$, the density of linear momentum, $g_i$, and the macroscopic polarization, $P_i$.

The presence of impurities, contaminants, etc., can be taken care of by adding a concentration variable *c*. If this concentration is conserved, one has thus an additional conserved quantity in the list of macroscopic variables. If this concentration is not conserved, as it will be typically the case for charged impurities such as ions, one will have a dynamic equation with a relaxation time contribution. For fast relaxation times, the associated equation will be no longer kept on the list of macroscopic variables, while for sufficiently long relaxation times its dynamic equation will be incorporated.

The first law of thermodynamics relates changes of the variables to changes of the energy density $\varepsilon$ by the Gibbs relation [18, 30].

1 $$\begin{aligned} d\varepsilon= & {} T\, d\sigma + \mu \, d\rho + \Pi \, d\phi + v_i dg_i + m_i dw_i \nonumber \\&+ h_i^P d{\hat{p}}_i + h^P dP + E_i d D_i \end{aligned}$$

where we have split the macroscopic polarization $P_i$ into its magnitude, *P*, and its direction, ${\hat{p}}_i$, where ${\hat{p}}_i$ is a polar unit vector meaning $({\hat{p}}_i)^2 = 1$; thus, $P_i = P {\hat{p}}_i$. The Einstein summation convention is used throughout the paper, whenever possible. The Gibbs relation contains the entropy density $\sigma$, representing the thermal degree of freedom, with its thermodynamic conjugate, the temperature *T*. Other conjugates are the chemical potential $\mu$, the osmotic pressure $\Pi$, the mean velocity $v_i = g_i / \rho$, $m_i$, the conjugate field to $w_i$, the molecular field associated with the polar unit vector ${\hat{p}}_i$, $h_i^P$, and the molecular field $h^P$ associated the magnitude of the polarization, *P*. Although we will not consider the electric charge density, nor $D_i$, the electric displacement field as independent variables, the contribution $E_i d D_i$ is necessary to be able to describe Maxwell stresses and field-induced pressure changes [18]. The conjugate $E_i$ is the local electric field containing internal and external contributions. Our notation follows closely that of Refs. [13, 29].

In the nonpolar case, the polar order is replaced by the uniaxial nematic order $(S/2)({\hat{n}}_i {\hat{n}}_j - (1/3) \delta _{ij})$, where the nematic director ${\hat{n}}_i$ describes the broken rotational symmetry. A director is part of the nematic order parameter and is therefore subject to the ${\hat{n}}_i \rightarrow -{\hat{n}}_i$ invariance. This restriction is absent for the polar case, where the polar order parameter $P {\hat{p}}_i$ contains the vector ${\hat{p}}_i$. To switch to the nonpolar case, one has to replace $h_i^P d{\hat{p}}_i + h^P dP$ by $h_i^n d{\hat{n}}_i + h^S dS$.

### Statics

The static behavior of the macroscopic system studied here is conveniently described by the energy functional in harmonic approximation Refs. [29, 31] including the kinetic energy densities

2 $$\begin{aligned} \varepsilon= & {} {\frac{1}{2} P_0 E } \,(\delta {\hat{p}}_i)^2 + { \frac{1}{2} c_P}(\delta P)^2 + \frac{1}{2}K_{ij}^{(2)}(\nabla _i P)(\nabla _j P) \nonumber \\&\quad + \frac{1}{2}K_{ijkl}(\nabla _i {\hat{p}}_j)(\nabla _k {\hat{p}}_l) + K_{ijk}^{(3)} (\nabla _i P)(\nabla _j {\hat{p}}_k) \nonumber \\&\quad + \frac{1}{2}c_{\rho \rho } (\delta \rho )^2 + \frac{1}{2}c_{\sigma \sigma }(\delta \sigma )^2 + \frac{1}{2}c_{\phi \phi }(\delta \phi )^2 \nonumber \\&\quad + c_{\rho \phi }(\delta \rho )(\delta \phi ) + c_{\rho \sigma }(\delta \rho )(\delta \sigma ) +c_{\sigma \phi }(\delta \sigma )(\delta \phi ) \nonumber \\&\quad + (\gamma _1 \delta \rho + \gamma _2 \delta \sigma + \gamma _3 \delta \phi ) \,\delta P \nonumber \\&\quad + (\theta _1 \delta \rho + \theta _2 \delta \sigma + \theta _3 \delta \phi ) \, {\hat{p}}_i \nabla _i P \nonumber \\&\quad + ({\bar{\theta }}_1 \delta \rho + {\bar{\theta }}_2 \delta \sigma + \bar{\theta }_3 \delta \phi ) \,\mathrm{div} {\varvec{{\hat{p}}}} + \frac{1}{2\rho } {\varvec{g}}^2 + \frac{1}{2} \alpha {\varvec{w}}^2 \quad \end{aligned}$$

where $\delta$ denotes deviations from the equilibrium value, in particular $\delta P = P - P_0$, $\delta {\hat{p}}_i = {\hat{p}}_i - {\hat{p}}_i^0$, $\delta \phi = \phi - \phi _0$. We only consider a spatially homogeneous ground state, meaning $P_0$, $\phi _0$ and ${\hat{p}}_i^0$ are constant and the direction of ${\hat{p}}_i^0$ is arbitrary.

Applying a constant external electric field $E_i$ with magnitude *E*, ${\hat{p}}_i^0$ will be parallel to the external field, ${\hat{p}}_i^0 = E_i / E$. The polarization electric coupling, $- {\varvec{P\cdot E}}$, translates into the hydrodynamic electric orientation energy $P_0 E (\delta {\hat{p}}_i)^2$ using ${\hat{p}}_i^0 \delta {\hat{p}}_i = -\frac{1}{2} (\delta {\hat{p}}_i)^2$. In addition, $P_0$ acquires an additional contribution linear in *E*, $\delta P_0 = \chi E$, with $\chi$ the electric susceptibility. In the following, we will use $P_0$ as short-hand notation also for the field case. The stiffness of order parameter variations is given by ${c_P}$.

Although the energy density expression is given in harmonic approximation only, it can give rise to nonlinear effects, since material parameters generally are still functions of the state variables, like temperature, pressure, and polarization $P_0$, and therefore also of *E*. This is in contrast to ordinary nematics, where the material parameters can only be a function of $E^2$.

Inhomogeneous deviations of the polarization are described by energy contributions isomorphic to that of a usual nematic phase including spatial modulations of the order parameter modulus. They comprise the Frank orientational elastic energy ($\sim K_{ijkl}$ with splay, bend and twist [32]), the energy associated with gradients of the modulus ($\sim K_{ij}^{(2)}$ of the standard uniaxial form) [18] and a cross-coupling term between gradients of the preferred direction to gradients of the order parameter modulus ($K_{ijk}^{(3)} =K^{(3)} (\delta _{ik}^\bot {\hat{p}}_j + \delta _{jk}^\bot {\hat{p}}_i)$ with $\delta _{jk}^\bot \equiv \delta _{jk} - {\hat{p}}_j {\hat{p}}_k$) [33].

In addition, there is the energy density of a fluid binary mixture in the third and fourth lines. In the fifth line, there are couplings ($\sim \gamma _n$) between the polarization and variations of $\rho$, $\sigma$ and $\phi$, which are of the same nature as the pyroelectric term in solids [34]. Other cross-coupling terms, $\sim \theta _{1,2,3}$ (line six) and $\sim \bar{\theta }_{1,2,3}$ (line seven), are relating variations of $\rho$, $\sigma$ and $\phi$ to splay, $\mathrm{div} {\varvec{{\hat{p}}}}$, and to spatial variations of the polarization along the preferred direction, ${\hat{p}}_i \nabla _i P$, respectively. We note that all these contributions are absent for nonpolar nematics, since they violate the $\hat{n}_i \rightarrow - \hat{n}_i$ invariance.

It is well known that a phase with $\mathrm{div} \,{\varvec{{\hat{p}}}} = const.$ (“splay phase”) does have a lower Ginzburg–Landau free energy (compared to the homogeneous state), but necessarily involves defects that increase the energy. The stability of such a splay phase depends, for example, on boundary conditions and will not be considered here. For a hydrodynamic treatment of splay phases, see [35]. Since we are dealing with a stable homogeneous equilibrium state here, the linear surface term, $\sim \mathrm{div} \,{\varvec{{\hat{p}}}}$, can be neglected.

Ordinary nonpolar nematics with a director field, $\mathbf {\hat{n}}$, show flexoelectricity described by a contribution to the generalized energy of the form (for $\mathrm{curl} \;{\varvec{E}}=0$) [18, 32] $\sim (\delta _{ij}^{\bot } n_k - \delta _{jk}^{\bot } n_i) (\nabla _i n_j) E_k$. For a polar system, the flexoelectric energy would read $\sim {\hat{p}}_i E_i (\mathrm{div} \,{\varvec{{\hat{p}}}})$ and simply renormalizes the prefactor of the linear splay term, which we neglect anyhow.

Finally, the kinetic energy $(1/2\rho _p) ({\varvec{g}}^{p})^2 \!+\! (1/2\rho _s) ({\varvec{g}}^{s})^2$ expressed by the momentum densities for the polar and the solvent subsystem, respectively, leads to $\alpha = \phi (1-\phi ) \rho$, since ${\varvec{g}}^{p} + {\varvec{g}}^{s} = {\varvec{g}}$.

Naturally the harmonic approximation is a restriction in the sense that only sufficiently small deviations from the spatially homogeneous ground state are contained. Big changes such as, for example, a complete director reorientation as in the Frederiks transition [32], require a fully nonlinear analysis of all the variables involved.

In the following, we list the expressions for the conjugated variables in terms of the hydrodynamic and macroscopic variables. They are defined as partial derivatives with respect to the appropriate variable, while all the other variables are kept constant, denoted by ellipses in the following

3 $$\begin{aligned} h^{'P}= & {} \frac{\partial \varepsilon }{\partial P}\big \arrowvert _{\dots } = {c_P} \delta P + \gamma _1 \delta \rho + \gamma _2 \delta \sigma + \gamma _3 \delta \phi \end{aligned}$$

4 $$\begin{aligned} \Phi ^{P}_{i}= & {} \frac{\partial \varepsilon }{\partial (\nabla _j P)}\big \arrowvert _{\dots } = K_{ij}^{(2)}(\nabla _j P) + K_{ijk}^{(3)} (\nabla _j {\hat{p}}_k) \nonumber \\&\quad \quad \quad \quad \quad \quad + (\theta _1 \delta \rho + \theta _2 \delta \sigma + \theta _3 \delta \phi ){\hat{p}}_i \quad \end{aligned}$$

5 $$\begin{aligned} h^{'P}_i= & {} \frac{\partial \varepsilon }{\partial {\hat{p}}_i}\big \arrowvert _{\dots } = { P_0 E}\, \delta {\hat{p}}_i \end{aligned}$$

6 $$\begin{aligned} \Phi _{ij}^{ P}= & {} \frac{\partial \varepsilon }{\partial (\nabla _j {\hat{p}}_i)}\big \arrowvert _{\dots } = K_{jikl}(\nabla _k {\hat{p}}_l) + K_{kji}^{(3)}(\nabla _k P) \nonumber \\&\quad \quad \quad \quad \quad \quad + ({\bar{\theta }}_1 \delta \rho +\bar{\theta }_2 \delta \sigma +{\bar{\theta }}_3 \delta \phi ) \delta _{ij}^{\bot } \quad \end{aligned}$$

7 $$\begin{aligned} \delta \mu= & {} \frac{\partial \varepsilon }{\partial \delta \rho }\big \arrowvert _{\dots } = \gamma _1 \delta P + \theta _1 {\hat{p}}_i \nabla _i P + {\bar{\theta }}_1 \mathrm{div} {\varvec{{\hat{p}}}} \nonumber \\&\quad \quad \quad \quad \quad + c_{\rho \rho }\delta \rho + c_{\rho \phi }\delta \phi + c_{\rho \sigma }\delta \sigma \nonumber \\&\quad \quad \quad \quad \quad + w_i^2 \phi (1 - \phi ) \end{aligned}$$

8 $$\begin{aligned} \delta T= & {} \frac{\partial \varepsilon }{\partial \delta \sigma }\big \arrowvert _{\dots } = \gamma _2 \delta P + \theta _2 {\hat{p}}_i \nabla _i P + {\bar{\theta }}_2 \mathrm{div} {\varvec{{\hat{p}}}} \nonumber \\&\quad \quad \quad \quad \quad + c_{\sigma \sigma }\delta \sigma + c_{\rho \sigma }\delta \rho + c_{\sigma \phi }\delta \phi \end{aligned}$$

9 $$\begin{aligned} \delta \Pi= & {} \frac{\partial \varepsilon }{\partial \delta \phi }\big \arrowvert _{\dots } = \gamma _3 \delta P + \theta _3 {\hat{p}}_i \nabla _i P + {\bar{\theta }}_3 \mathrm{div} {\varvec{{\hat{p}}}} \nonumber \\&\quad \quad \quad \quad \quad + c_{\phi \phi }\delta \phi + c_{\phi \rho }\delta \rho + c_{\phi \sigma }\delta \sigma \nonumber \\&\quad \quad \quad \quad \quad + w_i g_i + \rho w_i^2 (1 - 2 \phi ) \end{aligned}$$

10 $$\begin{aligned} m_i= & {} \frac{\partial \varepsilon }{\partial w_i}\big \arrowvert _{\dots } = \phi (1 - \phi ) \rho \,w_i \equiv \alpha \, w_i \end{aligned}$$

from which the total molecular fields, used in Eq. (1), $h^P = h^{'P} - \nabla _j\Phi ^P_{j}$ and $h_i^P = h^{'P}_i- \nabla _j\Phi _{ij}^{P}$ follow immediately. The $w_i$-dependence of the chemical potential and the osmotic pressure are due to the $\rho$- and $\phi$-dependence of $\alpha$.

### Dynamics

In the following, we will disregard electric field-induced dynamic effects, assuming only moderate field strengths. The dynamic equations have the form:

11 $$\begin{aligned} {\dot{\epsilon }} + \nabla _i (\epsilon + p) v_i + \nabla _i \bigl ( j_i^{\,\epsilon {\mathrm {R}}} + j_i^{\,\epsilon {\mathrm {D}}} \bigr )= & {} 0 , \end{aligned}$$

12 $$\begin{aligned} {\dot{\sigma }} + \nabla _i (\sigma v_{i} + j_i^{\,\sigma {\mathrm R}} + j_i^{\,\sigma {\mathrm D}})= & {} \frac{2R}{T},\quad \quad \end{aligned}$$

13 $$\begin{aligned} {\dot{\rho }} + \nabla _i (\rho v_i)= & {} 0 , \end{aligned}$$

14 $$\begin{aligned} \dot{g}_i + \nabla _j (g_i v_j + p \, \delta _{ij} + \sigma _{ij}^{\mathrm {th}} + \sigma _{ij}^{\, \mathrm {R}} + \sigma _{ij}^{\,\mathrm {D}} )= & {} 0 , \quad \end{aligned}$$

15 $$\begin{aligned} {\dot{\phi }} + v_j \nabla _j \phi + \frac{1}{\rho } \nabla _i m_i + \nabla _i ( j_i^{\phi \mathrm {R}} + j_i^{\phi \mathrm {D}})= & {} 0 , \end{aligned}$$

16 $$\begin{aligned} \dot{w}_i + v_j \nabla _j w_i + \nabla _i (\rho ^{-1} \Pi )+ X_i^{w \mathrm {R}} + X_i^{w \mathrm {D}}= & {} 0, \end{aligned}$$

17 $$\begin{aligned} \dot{P} + v_i \nabla _i P + X^{P \mathrm {R}} + X^{P \mathrm {D}}= & {} 0, \end{aligned}$$

18 $$\begin{aligned} \dot{p}_i + v_j \nabla _j {\hat{p}}_i + ({\varvec{{\hat{p}}}} \times {\varvec{\omega }})_i + X_i^{P \mathrm {R}} + X_i^{P \mathrm {D}}= & {} 0 \end{aligned}$$

19 $$\begin{aligned} {\dot{\rho }}_e + \nabla _j (\rho _e v_j)= & {} 0 , \end{aligned}$$

The conserved quantities and the entropy density contain phenomenological currents ($\sim j_i$), while the quasi-currents ($\sim X$) are associated with spontaneously broken continuous symmetry variables or macroscopic variables.

We use the vorticity $\omega _i = (1/2)\epsilon _{ijk}\nabla _j v_k$, the pressure *p* including the isotropic part of the Maxwell stress

20 $$\begin{aligned} p = \frac{\partial \,( \int \!\varepsilon dV )}{ \partial V}= -\varepsilon + \mu \rho + T\sigma + {\varvec{ v\cdot g}} + D_i E_i\nonumber \\ \end{aligned}$$

and the off-diagonal terms of the Maxwell and the Eriksen-type stresses [36]

21 $$\begin{aligned} 2 \sigma ^{\mathrm {th}}_{ij}= & {} - \left( E_iD_j + D_iE_j\right) + \Phi _j^P \nabla _i P + \Phi _i^P \nabla _j P \nonumber \\+ & {} \Phi _{kj}^{ P} \nabla _i {\hat{p}}_k + \Phi _{ki}^{ P} \nabla _j {\hat{p}}_k + \nabla _{k}({\hat{p}}_{j}\Phi _{ik}^P - {\hat{p}}_{i}\Phi _{jk}^P). \quad \quad \nonumber \\ \end{aligned}$$

The Maxwell stress is of the standard form [37, 38] and has been symmetrized with the help of the requirement that the energy density should be invariant under rigid rotations [18]. In detail, one first obtains directly from the condition of zero entropy production in Eq. (24)

22 $$\begin{aligned} \sigma ^{\mathrm {th}}_{ij}= & {} - D_j E_i + \Phi _j^P \nabla _i P + \Phi _{kj}^P \nabla _i {\hat{p}}_k \end{aligned}$$

and uses the requirement of rotational invariance of the Gibbs relation [15]

23 $$\begin{aligned} 0 = \epsilon _{ijk} (\Phi ^P_{jl} \nabla _l {\hat{p}}_k + \Phi ^P_{lj} \nabla _k {\hat{p}}_l) \end{aligned}$$

Compare also Ref. [15] for a detailed exposition.

The source term of Eq. (12) contains *R*, the dissipation function, which represents the energy dissipation of the system. Due to the second law of thermodynamics, *R* must satisfy $R\ge 0$: For reversible processes, this dissipation function is equal to zero, while for irreversible processes it must be positive

24 $$\begin{aligned} R= & {} -j_i^{\sigma *} \nabla _i T - j_{i}^{\phi *} \nabla _{i} \Pi - \sigma _{ij}^{*} \nabla _j v_i \nonumber \\&+ m_i \, X_i^{w*} + h_i^P \delta _{ik}^{\bot } X_k^{P*} + h^P X^{P*} \ge 0 \end{aligned}$$

where the upper sign applies for $* = D$ and the lower one for $*=R$.

The phenomenological currents and quasi-currents are the sum of the reversible and the dissipative part, as can be seen in Eqs. (11)–(18). The various transport contributions in Eqs. (11)–(18) (as well as *p* and $\sigma ^{\mathrm {th}}_{ij}$) are reversible and add up to zero in the entropy production.

These phenomenological currents and quasicurrents are treated in the following subsections within ’linear irreversible thermodynamics’ (guaranteeing general Onsager relations), i.e., as linear relations between currents and thermodynamic forces. The resulting expressions are nevertheless nonlinear, since all material parameters can be functions of the scalar state variables (e.g., *p*, *T*, *P*, $\phi$).

The form of Eq. (15) reflects the fact that both densities, $\rho _p$ and $\rho _s$, are conserved individually, and Eq. (17) describes the polar order parameter modulus as a slowly relaxing quantity (similar to, e.g., the nematic order parameter modulus [39] or the superfluid order [23, 40]). Although the electric charge density is not an independent degree of freedom, Eq. (19) is necessary to allow for the Maxwell stress [18].

In Eqs. (11)–(18), the transport and convective dynamic contributions are written in terms of the mean velocity, $v_i$. This guarantees compatibility with the general thermodynamic laws. In a two-fluid system, however, there are additional contributions of the transport and convective type in the reversible phenomenological currents that effectively modify transport and convective velocities (cf. the following section). In particular, it allows to describe specific models, where, for example, variables of the first (second) subsystem are transported and convected with the first (second) velocity. In this manuscript, we will not particularly focus on this point and refer to appropriate previous discussions [8, 10, 13].

### Reversible currents

To obtain the reversible currents, one expands all currents and quasi-currents systematically into the thermodynamic forces/conjugates taking into account the behavior under time reversal, spatial inversion, rigid rotations and, most importantly, zero entropy production. For a more detailed exposition of the method, we refer to Ref. [18]. For the reversible dynamic behavior of our macroscopic system, we obtain the following expressions for the reversible currents containing phenomenological parameters

25 $$\begin{aligned} j_i^{\,\sigma \mathrm {R}}= & {} {\bar{\beta }}_{ij} \,m_j + \varphi _{ijk}^{\sigma } A_{jk} + \varphi _{ijk}^{w \sigma } \nabla _j m_k, \end{aligned}$$

26 $$\begin{aligned} \sigma _{ij}^{\, \mathrm {R}}= & {} 2 \beta _2 \,m_i \,w_j + \lambda _{ij}^P h^P - \lambda _{kji} h_k^P \nonumber \\&\quad - \varphi _{kji}^\sigma \nabla _k T - \varphi _{kji}^\phi \nabla _k \Pi \end{aligned}$$

27 $$\begin{aligned} X_i^{w \mathrm {R} }= & {} {\bar{\beta }}_{ij} \nabla _j T + \gamma _{ij} \nabla _j \Pi +\beta _2 \,w_j (\nabla _i v_j + \nabla _j v_i) \nonumber \\&\quad + \beta _3 m_j (\nabla _j w_i - \nabla _i w_j) + \beta _{4} w_{j} (\nabla _{j} v_{i}- \nabla _{i} v_{j}) \nonumber \\&\quad + \nabla _j ( {\hat{\beta }}_{ji} h^P) - \beta _1 h_j^P \nabla _i p_j - \nabla _j (\lambda _{kji}^{m} h_k^P) \nonumber \\&\quad + \beta _5 h^P \nabla _i P + \varphi _{kji}^{w \sigma } \nabla _k \nabla _j T + \varphi _{kji}^{w \phi } \nabla _k \nabla _j \Pi \end{aligned}$$

28 $$\begin{aligned} X_i^{P \mathrm {R} }= & {} - \lambda _{ijk} \nabla _j v_k - \lambda _{ijk}^{m} \nabla _j m_k + \beta _1 \,m_j \nabla _j\,p_i , \end{aligned}$$

29 $$\begin{aligned} X^{P \mathrm {R}}= & {} \beta _{ij} A_{ij} + \hat{\beta }_{ij} \nabla _i m_j - \beta _5 m_i \nabla _i h^P , \end{aligned}$$

30 $$\begin{aligned} j_i^{\phi \mathrm {R}}= & {} \gamma _{ij} \,m_j + \varphi _{ijk}^{\phi } A_{jk}, + \varphi _{ijk}^{w \phi } \nabla _j m_k, \end{aligned}$$

with $\lambda _{ijk}^{m} = \frac{1}{2} \lambda _1^{m} \delta _{ij}^{\bot } {\hat{p}}_k +\frac{1}{2} \lambda _2^{m} \delta _{ik}^{\bot } {\hat{p}}_j$, $\lambda _{ijk}$ given by Eq. (31). The coupling of the polarization and the density of linear momentum are provided by the tensors

31 $$\begin{aligned} \lambda _{ijk} = \lambda ({\hat{p}}_j \delta _{ik}^{\bot } + {\hat{p}}_k \delta _{ij}^{\bot }) \,\,\, \mathrm{and} \,\,\, \lambda _{ij}^P = \lambda ^P_2 \delta _{ij}^{\bot } + \lambda ^P_3 {\hat{p}}_i {\hat{p}}_j \,\,\, \end{aligned}$$

One finds a total of three material-dependent coupling terms. The first is the analogue of the classical flow alignment term coupling the orientation of the preferred direction, ${\hat{p}}_i$, to deformational flow, while the coupling to rotational flow (rigid rotation) is not material dependent and has already been made explicit in Eq. (18). The two contributions $\sim \lambda ^P_2$ and $\sim \lambda ^P_3$ are associated with the coupling of the magnitude of the polarization, *P*, to velocity gradients. For the reversible coefficients $\beta _1, \dots , \beta _5$ as well as for the tensors ${\bar{\beta }}_{ij}$ and ${\hat{\beta }}_{ij}$, we follow closely the notation of Refs. [8, 9] and [13]. Finally we have for $\varphi _{ijk}^{\alpha }$ and $\varphi _{ijk}^{w \alpha }$ the structure

32 $$\begin{aligned} \varphi _{ijk}^{\alpha } = \varphi ^{\alpha }_1 {\hat{p}}_i {\hat{p}}_j {\hat{p}}_k + \varphi ^{\alpha }_2 {\hat{p}}_i \delta _{jk}^{\bot } + \varphi ^{\alpha }_3 \left( {\hat{p}}_j \delta _{ik}^{\bot } + {\hat{p}}_k \delta _{ij}^{\bot }\right) \qquad \end{aligned}$$

where $\alpha \in \{\sigma , \Pi \}$. These reversible dynamic cross-coupling terms exist in all macroscopic systems with a parity breaking vector (compare, for example, Ref. [41]). Naturally the terms $\sim \varphi _{ijk}^{w \,\alpha }$ exist only for two-fluid systems, since there is no relative velocity otherwise.

In Eqs. (25)–(30), all contributions with any third-rank tensor $\varphi _{ijk}$ are odd in ${\hat{p}}_i$ and therefore absent in nonpolar nematics.

### Dissipative currents

To describe dissipative processes, it is convenient to expand the dissipation function, *R*, the source term in the dynamic equation for the entropy density, into an expression quadratic in the thermodynamic forces, which is positive. Then, taking variational derivatives (or partial derivatives when applicable) of *R* with respect to forces, one obtains linear relations between the currents and the quasi-currents on the one hand and thermodynamic forces on the other (see also below). The entropy production is a scalar under all transformations compatible with symmetry including time reversal, spatial parity, and rigid rotations. The positivity of *R* guarantees automatically that inequality (24) is satisfied for dissipative contributions. For a detailed exposition of the method, we refer to Ref. [18]. The dissipative dynamic behavior of our macroscopic system is characterized by the dissipation function *R*

33 $$\begin{aligned} R= & {} \kappa _{ij} (\nabla _i T) (\nabla _j T) + D_{ij} (\nabla _i \Pi )(\nabla _j \Pi ) \nonumber \\&+ 2D^{T \phi }_{ij} (\nabla _i T)(\nabla _j \Pi ) \nonumber \\&+ \xi _{ij} m_i m_j + \nu _{ijkl} (\nabla _j v_i)(\nabla _l v_k) \nonumber \\&+ 2 \nu _{ijkl}^{\,c} (\nabla _j v_i)(\nabla _l m_k) + \nu _{ijkl}^{\,w} (\nabla _j m_i)(\nabla _l m_k) \nonumber \\&+ b_{\bot } h_i^P h_i^P + b_{||} h^{P} h^{P} \nonumber \\&+ 2\kappa ^{P}_{\bot } \delta _{ij}^{\bot } (\nabla _i T) h_j^P + 2\kappa ^{P}_{||}({\hat{p}}_i \nabla _i T) h^{P} \nonumber \\&+ 2D^{P}_{\bot } \delta _{ij}^{\bot } (\nabla _i \Pi ) h_j^P + 2D^{P}_{||}({\hat{p}}_i \nabla _i \Pi )h^{P} \end{aligned}$$

The tensors $\kappa _{ij}$, $D^{T\phi }_{ij}$, $D_{ij}$, $\xi _{ij}$ as well as $\nu _{ijkl}$ and $\nu _{ijkl}^{w}$ are of the standard uniaxial form for second and fourth ranks tensors [18, 34]. The tensor $\nu _{ijkl}^{c}$ lacks the $\nu _{ijkl} \leftrightarrow \nu _{klij}$ invariance and therefore has one coefficient more than $\nu _{ijkl}$ [34]. The contribution $\sim b_{||}$ in the entropy production describes the relaxation of the polarization modulus *P*, while the contribution associated with $b_{\bot }$ corresponds to the diffusion of the preferred direction (conventionally called $\gamma _1^{-1}$ in the literature of nematodynamics). These terms have their analogues in ordinary nematics (with the order parameter modulus included). Specific for polar nematics and polar nematics with a solvent are the dissipative cross-couplings between polarization and gradients of temperature and osmotic pressure governed by the material parameters $\kappa ^P_{\bot }$, $\kappa ^P_{\parallel }$ as well as $D^P_{\bot }$, $D^P_{\parallel }$.

The four last contributions with coefficients $\kappa _{\perp ,\parallel }^P$ and $D_{\perp ,\parallel }^P$ cannot exist in nonpolar nematics, since they are odd in ${\hat{p}}_i$.

To obtain the dissipative parts of the currents and quasicurrents, we take the partial derivatives of *R* with respect to the appropriate thermodynamic force

34 $$\begin{aligned} j^{\sigma \mathrm {D}}_i= & {} - \frac{\partial R}{\partial (\nabla _i T)}\big \arrowvert _{\dots } = - \kappa _{ij}(\nabla _jT) - D^{T\phi }_{ij}(\nabla _j\Pi ) \nonumber \\&\quad \quad \quad \quad \quad \quad \quad - \kappa ^P_{\bot } h_i^P - \kappa ^P_{||} {\hat{p}}_i h^P \end{aligned}$$

35 $$\begin{aligned} j^{\phi \mathrm {D}}_i= & {} - \frac{\partial R}{\partial (\nabla _j\Pi )}\big \arrowvert _{\dots } = - D_{ij}(\nabla _j\Pi ) - D^{T\phi }_{ij}(\nabla _j T) \nonumber \\&\quad \quad \quad \quad \quad \quad \qquad - D^{P}_{\bot } h_i^P - D^P_{||} {\hat{p}}_i h^P \end{aligned}$$

36 $$\begin{aligned} \sigma ^{\mathrm {D}}_{ij}= & {} - \frac{\partial R}{\partial (\nabla _jv_i)}\big \arrowvert _{\dots } = - \nu _{ijkl}A_{kl} - \nu _{ijkl}^{c} (\nabla _l m_k) \quad \quad \end{aligned}$$

37 $$\begin{aligned} X_i^{w \mathrm {D}}= & {} \frac{\delta R}{\delta m_i}\big \arrowvert _{\dots } = \xi _{ij} m_j \nonumber \\&\quad \quad \quad \qquad - \nabla _j ( \nu _{ijkl}^{w} \nabla _l m_k + \nu _{ijkl}^{c} A_{kl}) \end{aligned}$$

38 $$\begin{aligned} X_i^{P \mathrm {D}}= & {} \frac{\partial R}{\partial h_i^P}\big \arrowvert _{\dots } = b_{\bot } h_i^P \nonumber \\&\quad \quad \quad \qquad + \delta _{ij}^{\bot } ( D^P_{\bot } \nabla _j \Pi + \kappa ^{P}_{\bot } \nabla _j T) \end{aligned}$$

39 $$\begin{aligned} X^{P \mathrm {D}}= & {} \frac{\partial R}{\partial h^P}\big \arrowvert _{\dots } = b_{||} h^P \nonumber \\&\quad \quad \quad \qquad + {\hat{p}}_i (D^P_{||} \nabla _i \Pi + \kappa ^{P}_{||} \nabla _i T) \end{aligned}$$

## Two-fluid model for polar nematics with a chiral solvent-polar cholesterics

In this section, we consider a two-fluid system composed of a polar nematic phase and a chiral liquid component. The analysis applies equally well to a polar cholesterics phase in an isotropic solvent as long as the bulk of the phase is considered. Compared to the system of the preceding section, a polar nematic phase with an isotropic solvent, the additional feature is now the existence of chirality. The latter is described by a pseudoscalar quantity, $q_0$, that is invariant under proper rotations, but changes sign if a spatial inversion is involved, thereby breaking inversion symmetry. Although inversion symmetry is already broken by the polar preferred direction, the pseudoscalar quantity leads to additional effects. In an isotropic liquid chirality leads to optical activity, the rotation of the plane of linearly polarized light, while in liquids with a preferred direction, e.g., cholesterics, this preferred direction orients in a helical fashion in the ground state. The same can be expected for chiral polar nematics.

In the present case, chirality is a molecular property, either of the solvent or of the polar cholesterics (or both). It is taken as a given property of the physical system considered. Since the 2-fluid system is mixed on the microscopic level, chirality applies to all degrees of freedom, even those of the nonchiral subsystem. Thus, the macroscopic variables are the same as in the achiral case and the Gibbs relation Eq. (1) as well as the form of the dynamic Eqs. (11)–(19) can be taken over. That means, we will use the ‘local description’ [32, 42], starting from a homogeneous ground state, and describe the chiral effects by adding all possible contributions, linear in $q_0$, to the energy density (statics) and the phenomenological currents (reversible and irreversible dynamics).

In the statics, there are the following chiral contributions

40 $$\begin{aligned} \varepsilon _{q_0}= & {} \dots + {\tilde{K}}_2 q_0 \mathbf{({\hat{p}} \cdot \mathrm{curl}\,{\hat{p}})} + K_7 q_0 \mathbf{({\hat{p}} \cdot \mathrm{curl}\,{\hat{p}})\, \mathrm{div}\, {\hat{p}}} \nonumber \\&\quad + q_0 (\alpha _1 \delta \rho + \alpha _2 \delta \sigma + \alpha _3 \delta \phi + \alpha _4 \delta P) (\mathbf{{\hat{p}} \cdot \mathrm{curl}\,{\hat{p}}}) \nonumber \\ \end{aligned}$$

where the dots denote the nonchiral contributions given in Eq. (2). There is the linear twist term (${\tilde{K}}_2$) well known from nonpolar chiral nematics, which gives rise to a helical equilibrium structure $({ \mathbf {\hat{p}} \cdot \mathrm{curl}\,{\hat{p}}} )_0= q_0 {\tilde{K}}_2 / K_2$, with $K_2$ the Frank-like modulus for the quadratic, nonchiral twist energy. In nonpolar nematics, very often ${\tilde{K}}_2 = K_2$ is assumed, although there is no a priori reason to do so and in the early discussions the two moduli are indeed discriminated [43, 44].

The second contribution ($\sim K_7$) is a coupling between twist and splay, which does not have a counterpart in nonpolar cholesterics. The last line ($\sim \alpha _{1,2,3,4}$) describes couplings between twist and variations of the scalar variables giving rise to the static Lehmann effect as in the nonpolar case [42].

For the reversible parts of the currents we find, requiring vanishing entropy production, *R*, and indicating the terms already present for polar nematics by $\dots$

41 $$\begin{aligned} j_i^{\sigma \mathrm {R}}= & {} \dots + \Gamma _2 q_0 (s_{jik} + s_{kij}) A_{jk} \nonumber \\&+ \Gamma _4 q_0 (s_{jik} + s_{kij}) \nabla _j m_k , \end{aligned}$$

42 $$\begin{aligned} \sigma _{ij}^{\, \mathrm {R}}= & {} \dots - \Gamma _2 q_0 (s_{ikj}+s_{jki}) \nabla _k T \nonumber \\&- \Gamma _3 q_0 (s_{ikj} + s_{jki}) \nabla _k \Pi \nonumber \\&- \Gamma _1 q_0 (s_{ikj} + s_{jki}) h_k^P \end{aligned}$$

43 $$\begin{aligned} X_i^{w \mathrm {R}}= & {} \dots + \Gamma _4 q_0 (s_{jik} + s_{kij}) \nabla _j \nabla _k T \nonumber \\&+\Gamma _5 q_0 (s_{jik} + s_{kij}) \nabla _j \nabla _k \Pi \nonumber \\&+ q_0 (\Gamma _{6a} s_{jik} + \Gamma _{6b} s_{kij}) \nabla _j h^P_k , \end{aligned}$$

44 $$\begin{aligned} j_i^{\phi \mathrm {R}}= & {} \dots + \Gamma _3 \,q_0 (s_{jik} + s_{kij}) A_{jk} \nonumber \\&+ \Gamma _5 \,q_0 (s_{jik} + s_{kij}) \nabla _k \nabla _j m_i \end{aligned}$$

45 $$\begin{aligned} X_i^{P \mathrm {R}}= & {} \dots + \Gamma _1 q_0 (s_{jik} + s_{kij}) A_{jk} \nonumber \\&+ q_0 (\Gamma _{6a} s_{jik} + \Gamma _{6b} s_{kij}) \nabla _j m_k \end{aligned}$$

with $s_{ijk} ={\hat{p}}_i {\hat{p}}_m \epsilon _{mjk}$ and ${\hat{p}}_i$ the polar unit vector. There are no chiral contributions to $X^{PR}$.

The additional chiral reversible terms either involve gradients of the relative velocity ($\Gamma _4,\Gamma _5,\Gamma _{6a,b}$) or of the mean velocity ($\Gamma _1,\Gamma _2,\Gamma _3$) coupling to temperature, osmotic pressure and reorientation of the polar direction. They have not been considered before. In addition, we notice that the cross-coupling terms $\sim \Gamma _1$,$\sim \Gamma _2$ and $\sim \Gamma _3$ also exist in a one-fluid polar cholesteric phase.

The chiral reversible currents, involving $\Gamma _2,\Gamma _3,\Gamma _4,\Gamma _5$, are present in nonpolar nematics as well, since the tensors $s_{ijk}$ are even in ${\hat{p}}_i$. The contributions $\sim \Gamma _2$ and $\sim \Gamma _3$ have already been discussed for nonpolar one-fluid cholesterics in Ref. [32]. On the other hand, the contributions $\sim \Gamma _1$ and $\sim \Gamma _{6a,6b}$ are forbidden in nonpolar nematics, since the nematic current $X_i^{nR}$ as well as the nematic conjugate $h_i^n$ are subject to the $\hat{n}_i \rightarrow - \hat{n}_i$ invariance.

For the chiral dissipative contributions, we find

46 $$\begin{aligned} R= & {} \dots + \Sigma ^{\mathrm {pol}}\, q_0 (s_{jik} + s_{kij}) \,m_i A_{jk} \nonumber \\&\quad + q_0 ( \Psi ^T \nabla _i T + \Psi ^{\phi } \nabla _i \Pi +\Psi ^P \nabla _i h^P) \epsilon _{ijk} {\hat{p}}_k h_j^P \qquad \end{aligned}$$

where $\dots$ stands for the dissipative nonchiral contributions Eq. (33).

The most interesting dissipative cross-coupling is clearly the contribution $\sim \Sigma ^{pol}$ describing a direct cross-coupling between relative velocities and symmetrized gradients of the mean velocity. It does not exist for nonchiral polar nematics and requires for its existence a pseudoscalar as well as a preferred direction.

There are further dissipative coupling terms between the molecular field of the director, $h_i^P$, and temperature and concentration gradients, as well as the force associated with the polar order modulus, $h^P$. They are the dissipative parts of the Lehmann effect for a polar system—familiar from cholesteric and chiral smectic liquid crystals for nonpolar systems [42, 45–48]. All contributions to Eq. (46) survive the transfer to the nonpolar case.

From Eq. (46), we get the following chiral dissipative parts of the currents

47 $$\begin{aligned} \sigma _{ij}^{\, \mathrm {D}}= & {} - \Sigma ^{\mathrm {pol}} \,q_0 (s_{ikj} + s_{jki}) m_k, \end{aligned}$$

48 $$\begin{aligned} X_i^{w \mathrm {D}}= & {} + \Sigma ^{\mathrm {pol}} + q_0 (s_{jik} + s_{kij}) A_{jk} \end{aligned}$$

49 $$\begin{aligned} j_i^{\sigma \mathrm {D}}= & {} - q_0 \Psi ^T \epsilon _{ijk} \,{\hat{p}}_k h_j^P \end{aligned}$$

50 $$\begin{aligned} j_i^{\phi \mathrm {D}}= & {} - q_0 \Psi ^\phi \epsilon _{ijk} \,{\hat{p}}_k h_j^P \end{aligned}$$

51 $$\begin{aligned} X^{P \mathrm {D}}= & {} - q_0 \Psi ^ P \epsilon _{ijk} \,{\hat{p}}_k \nabla _i h_j^P \end{aligned}$$

52 $$\begin{aligned} X_i^{P \mathrm {D}}= & {} + q_0 ( \Psi ^T \nabla _j T + \Psi ^{\phi } \nabla _j\Pi +\Psi ^P \nabla _j h^P)\epsilon _{jik} \,{\hat{p}}_k \quad \end{aligned}$$

with $s_{ijk}$ defined after Eq. (45).

## Incorporation of a strain field for polar nematic gels and elastomers in a solvent

### Nonchiral solvent

In this first subsection, we discuss how the two-fluid macroscopic dynamics is modified when a strain field and relative rotations are incorporated for polar nematics to make the equations applicable to gels and elastomers. We will make extensive use of the macroscopic dynamics for 1-fluid polar nematic gels and elastomers [31].

The presence of a network in polar nematics gives rise to two additional macroscopic variables: The strain tensor $u_{ij}$ and relative rotations ${\tilde{\Omega }}_i$. The strain can be written in linearized form as $u_{ij} = \frac{1}{2} (\nabla _i u_j + \nabla _j u_i)$ with the displacement field $u_i$. For a nonlinear generalization cf. [49]. We will focus on the case of a permanently cross-linked gel or elastomer, where $u_{ij}$ does nor relax, but only diffuse.

Due to the simultaneous presence of a network as well as of the variables $\delta {\hat{p}}_i$, relative rotations (as pioneered by de Gennes [50] for nematic elastomers) become an important macroscopic variable, which can be introduced in a linear description via

53 $$\begin{aligned} {\tilde{\Omega }}_i = \delta {\hat{p}}_i - {\hat{p}}_j \Omega _{ij} \end{aligned}$$

with $\Omega _{ij} = \frac{1}{2} (\nabla _i u_j -\nabla _i u_i)$. For a nonlinear definition, cf. [51]. Relative rotations are perpendicular to the polar direction, ${\hat{p}}_i {\hat{p}}_{j} {\tilde{\Omega }}_{ij} = 0$. This variable describes the fact that in the presence of tensor (or other vector) fields, rotations of ${\hat{p}}_i$ do not cost energy, only if also those fields are rotated the same way, but cost energy otherwise. Relative rotations are not truly hydrodynamic variables, but relax slowly enough to be considered here.

The Gibbs relation Eq. (1) has to be modified accordingly

54 $$\begin{aligned} d\varepsilon= & {} \dots + \psi _{ij} d u_{ij} + L_i^{\bot } d {\tilde{\Omega }}_{i} \end{aligned}$$

where the dots denote all the contributions already given in Sect. 2. The additional thermodynamic conjugate quantities are the elastic stress $\psi _{ij}$ and the relative molecular field $L_i^{\bot }$ associated with relative rotations.

The strain and the relative rotations bring a host of additional contributions to the energy, Eq. (2). However, none is related to the 2-fluid situation. Therefore, we can refer to the 1-fluid expression (Eq. (3)ff of [31]) without copying it here.

For the dynamic equations, we have in addition

55 $$\begin{aligned} \dot{{\tilde{\Omega }}}_i +v_{j} \nabla _{j} {\tilde{\Omega }}_i + Y_i^{\Omega \mathrm {R}} + Y_i^{\Omega \mathrm {D}}= & {} 0 \end{aligned}$$

56 $$\begin{aligned} \dot{u}_{ij} +v_{j} \nabla _{j} u_{ij} - A_{ij} + X_{ij}^{u \mathrm {R}} + X_{ij}^{u \mathrm {D}}= & {} 0 \end{aligned}$$

while the other dynamic equation are of the same form as in Sect. 2. The nonlinear, nonphenomenological part of the stress tensor, $\sigma _{ij}^{th}$, Eq. (21), now takes the form

57 $$\begin{aligned} 2\sigma _{ij}^{\mathrm {th}}= & {} - ( E_{j}D_{i} + E_i D_j) + \Phi _j^P \nabla _i P + \Phi _i^P \nabla _j P \nonumber \\&\quad + \Phi _{kj}^{ P} \nabla _i {\hat{p}}_k + \Phi _{ki}^{ P} \nabla _j {\hat{p}}_k + \nabla _{k}({\hat{p}}_{j}\Phi _{ik}^P - {\hat{p}}_{i}\Phi _{jk}^P) \nonumber \\&\quad + 2 \psi _{jk} u_{ki} + 2 \psi _{ik} u_{kj} \end{aligned}$$

with the elasticity-related part symmetrized due to rotational invariance of the energy density.

For the nonchiral reversible currents, we have, in addition to the terms given in Sect. 2

58 $$\begin{aligned} \sigma _{ij}^{\mathrm {R}}= & {} - \frac{1}{2} \lambda ^{\bot } (L_i^{\bot } {\hat{p}}_j + L_j^{\bot } {\hat{p}}_i) \quad \end{aligned}$$

59 $$\begin{aligned} X_k^{w \mathrm {R}}= & {} - \Xi _{ijk} \psi _{ij} - \Lambda \delta _{kj}^{\bot } L_j^{\bot } \end{aligned}$$

60 $$\begin{aligned} X_{ij}^{u \mathrm {R}}= & {} \Xi _{ijk} m_k \end{aligned}$$

61 $$\begin{aligned} Y_{i}^{\Omega \mathrm {R}}= & {} -\frac{1}{2}\lambda ^{\bot } (\delta _{ij}^{\bot } {\hat{p}}_k + \delta _{ik}^{\bot } {\hat{p}}_j) A_{jk} + \Lambda \delta _{ij}^{\bot } m_j \end{aligned}$$

with

62 $$\begin{aligned} \Xi _{ijk} = \Xi _1 {\hat{p}}_i {\hat{p}}_j {\hat{p}}_k + \Xi _2 \delta _{ij}^{\bot } {\hat{p}}_k + \Xi _3 ( \delta _{ik}^{\bot } {\hat{p}}_j + \delta _{jk}^{\bot } {\hat{p}}_i)\qquad \end{aligned}$$

The contributions $\sim \lambda ^{\bot }$, involving relative rotations and mean velocity flow, already exist in a 1-fluid description. The couplings $\sim \Xi _{ijk}$ (relating elasticity and the velocity difference) and $\sim \Lambda$ (relating relative rotations and the velocity difference) are specific for the 2-fluid situation.

We note that the contribution $\sim \Xi _3$ has been given recently [52], where the authors have also elucidated its biological consequences in detail.

Of the reversible currents related to $u_{ij}$ and ${\tilde{\Omega }}_i$, Eqs. (58)–(61), only the contributions $\sim \lambda ^\bot$ are possible in the nonpolar case, while both cross-couplings to the relative velocity, $\sim \Xi _{ijk}$ and $\sim \Lambda$, are not, since $\sim \Xi _{ijk}$, $L_i^\bot$ and $Y_i^\Omega$ are all odd in ${\hat{p}}_i$.

For the entropy production *R*, governing the dissipative parts of the currents, we do not have any additional contributions due to the strain or the relative rotation degree of freedom, which are related to the 2-fluid situation. Thus, we refer again to the 1-fluid expression (Eq. (46) of [31]), which we will not duplicate here.

### Chiral solvent

In the present subsection, we add chirality to the system described in the preceding subsection. The existence of a pseudoscalar $q_0$ allows for the additional contributions to the energy [36], keeping in mind that we are dealing here with a polar system with a polar direction, ${\hat{p}}_i$, instead of a director, ${\hat{n}}_i$.

63 $$\begin{aligned} \varepsilon = \cdots - q_0 \tau _{ij}^u \,{\varvec{ {\hat{p}} \cdot }} ({\varvec{\nabla }} \times {\varvec{{\hat{p}}}}) \, u_{ij} - q_0 \tau _\Omega \epsilon _{ikm} {\hat{p}}_j {\hat{p}}_m {\tilde{\Omega }}_i \nabla _j {\hat{p}}_k \quad \quad \end{aligned}$$

where $\tau _{ij}^u$ is of standard uniaxial form. The dots represent the energy contributions from the previous sections. Both terms survive the transition to the nonpolar case, since ${\tilde{\Omega }}_i = \delta {\hat{n}}_i - {\hat{n}}_j \Omega _{ij}$ [53] is then differently defined. It should be noted that the only 2-fluid term is still the kinetic energy, $\sim \alpha w_i^2$ in Eq. (2).

For the reversible chiral currents we have, in addition to the terms given in Sect. 3

64 $$\begin{aligned} X_k^{w \mathrm {R}}= & {} - q_0 \,{\tilde{\Xi }}_{ijk} \,\psi _{ij} \end{aligned}$$

65 $$\begin{aligned} X_{ij}^{u \mathrm {R}}= & {} q_0 \,{\tilde{\Xi }}_{ijk} \,m_k \end{aligned}$$

where

66 $$\begin{aligned} {\tilde{\Xi }}_{ijk} = {\tilde{\Xi }} ( \epsilon _{ikm} {\hat{p}}_j {\hat{p}}_m + \epsilon _{jkm} {\hat{p}}_i {\hat{p}}_m) \end{aligned}$$

This coupling between the elastic degree of freedom and the relative velocity is specific for a 2-fluid description. It also exists in nonpolar nematics, since ${\tilde{\Xi }}_{ijk}$ is even in ${\hat{p}}_i$.

For the additional chiral dissipative contributions, we get

67 $$\begin{aligned} R= & {} \cdots + q_0\, \epsilon _{ijk} {\hat{p}}_j h_k^p \psi _{\phi } \nabla _i \Pi \nonumber \\&\quad + q_0 \, \epsilon _{ijk} {\hat{p}}_j L_k^{\bot } (\psi _{\phi }^\Omega \nabla _i \Pi +\psi _\sigma ^\Omega \nabla _i T ) \\&\quad + q_0 \psi _{jk} (\chi _{ijk}^{\phi \psi } \nabla _i \Pi + \chi _{ijk}^{\sigma \psi } \nabla _i T) \end{aligned}$$

where the dots represent contributions from previous sections. The material tensors $\chi _{ijk}^{\xi \psi }$, with $\xi \in \{\sigma , \phi \}$, contain one phenomenological parameter each

68 $$\begin{aligned} \chi _{ijk}^{\xi \psi } = \chi ^{\xi \psi } (\epsilon _{ikm} {\hat{p}}_j {\hat{p}}_m + \epsilon _{ijm} {\hat{p}}_k {\hat{p}}_m ) \end{aligned}$$

The dissipative dynamic contributions in Eq. (67) are also present in the nonpolar case, and at the same time also exist in a 1-fluid description, they have counterparts in the 1-fluid macroscopic dynamics of ferrocholesterics [36], if there, the nematic director ${\hat{n}}_i$ is replaced by ${\hat{p}}_i$.

## Possible dynamic experiments

### Reversible coupling terms in polar nematics

The reversible currents in polar nematics, Eqs. (25)–(30), contain cross-couplings between relative velocities and, e.g., the heat current

69 $$\begin{aligned} j_i^{\sigma \mathrm {R}} = \varphi _{ijk}^{w \sigma } \nabla _j m_k, \end{aligned}$$

with

70 $$\begin{aligned} \varphi _{ijk}^{w \sigma } = \varphi ^{w \sigma }_1 {\hat{p}}_i {\hat{p}}_j {\hat{p}}_k + \varphi ^{w \sigma }_2 {\hat{p}}_i \delta _{jk}^{\bot } + \varphi ^{w \sigma }_3 ({\hat{p}}_j \delta _{ik}^{\bot } + {\hat{p}}_k \delta _{ij}^{\bot })\qquad \end{aligned}$$

Taking the ${\hat{z}}$-axis as polar axis, one gets

71 $$\begin{aligned} j_x^{\sigma \mathrm {R}}= & {} \varphi ^{w \sigma }_3 (\nabla _z w_x + \nabla _x w_z) \end{aligned}$$

72 $$\begin{aligned} j_y^{\sigma \mathrm {R}}= & {} \varphi ^{w \sigma }_3 (\nabla _z w_y + \nabla _y w_z ) \end{aligned}$$

73 $$\begin{aligned} j_z^{\sigma \mathrm {R}}= & {} \varphi ^{w \sigma }_1 \nabla _z w_z + \varphi ^{w \sigma }_2 (\nabla _x w_x + \nabla _y w_y ) \end{aligned}$$

From Eqs. (71) and (72), we see that any pure shear flow of the relative velocity, in a plane that contains the preferred direction, leads to an in-plane heat current perpendicular to the preferred direction. From Eq. (73), we conclude that a heat flow along the preferred direction is induced by an extensional flow along the preferred direction and, with a different magnitude, also along any perpendicular direction.

The same analysis applies to a concentration current, Eq. (30) with $\varphi ^{w \phi }_{1,2,3}$. If one considers gradients of the mean velocity ($A_{ij}$), instead of the relative velocity, appropriate effects are found, but they are not specific for a two-fluid description.

The reciprocal effect in Eq. (27)

74 $$\begin{aligned} X_i^{w \mathrm {R}} = \varphi _{ijk}^{w \sigma } \nabla _j \nabla _k T \end{aligned}$$

leads to

75 $$\begin{aligned} X_x^{w \mathrm {R}}= & {} + \varphi ^{w \sigma }_3 \nabla _x \nabla _z T \end{aligned}$$

76 $$\begin{aligned} X_y^{w \mathrm {R}}= & {} + \varphi ^{w \sigma }_3 \nabla _y \nabla _z T \end{aligned}$$

77 $$\begin{aligned} X_z^{w \mathrm {R}}= & {} \varphi ^{w \sigma }_1 \nabla _z^2 T + \varphi ^{w \sigma }_2 {\varvec{\nabla }}_\bot ^2 T \end{aligned}$$

describing how second-order gradients of temperature (and concentration) lead to flow in the relative velocity.

Finally, we point out that all the effects described in this subsection are restricted to polar nematics and do not exist in nonpolar nematics.

### Reversible coupling terms in polar cholesterics

Inspecting the reversible coupling terms in Eqs. (41)–(45), some quite intuitive possibilities emerge to detect these contributions. Taking the heat current as an example, we get

78 $$\begin{aligned} j_i^{\sigma \mathrm {R}}= \dots + \Gamma _4 q_0 (s_{jik} + s_{kij}) \nabla _j m_k. \end{aligned}$$

with $$s_{ijk} = {\hat{p}}_i {\hat{p}}_m \epsilon _{mjk}$$

Taking the polar direction ${\hat{p}}_i$ parallel to the ${\hat{z}}$-direction, we have explicitly

79 $$\begin{aligned} j_x^{\sigma \mathrm {R}}= & {} \dots + \Gamma _4 q_0 (\nabla _z m_y + \nabla _y m_z) \end{aligned}$$

80 $$\begin{aligned} j_y^{\sigma \mathrm {R}}= & {} \dots - \Gamma _4 q_0 (\nabla _z m_x + \nabla _x m_z) \end{aligned}$$

Or in simple terms: the symmetrized gradient of the velocity difference in the $y-z$-plane leads to a heat current in $x-$direction and a symmetrized gradient of the velocity difference in the $x-z$-plane leads to a heat current of the same magnitude and the opposite sign in the $y-$direction.

The same analysis applies to a concentration current, Eq. (44) with $\Gamma _5$. If one considers gradients of the mean velocity ($A_{ij}$), instead of the relative velocity, appropriate effects are found, but they are not specific for a two-fluid description.

It should be noted that these couplings of the same variables as in the nonchiral case, Sect. 5.1, are geometrically more involved, in particular show twisting tendencies.

The counter terms in Eq. (43), necessary to have zero entropy production, lead to

81 $$\begin{aligned} X_x^{w \mathrm {R}}= & {} \dots + 2 \Gamma _4 q_0 \nabla _y \nabla _z T \end{aligned}$$

82 $$\begin{aligned} X_y^{w \mathrm {R}}= & {} \dots - 2 \Gamma _4 q_0 \nabla _x \nabla _z T \end{aligned}$$

meaning that a temperature field, bent in a plane, leads to a relative velocity current perpendicular to the bending plane.

Somewhat similar effects are described by the reversible transport coefficients $\Gamma _{6a}$ and $\Gamma _{6b}$ in Eqs. (43) and (45)

83 $$\begin{aligned} X_x^{w \mathrm {R}}= & {} \dots + q_0 \Gamma _{6a} \nabla _z h_y^P + q_0 \Gamma _{6b} \nabla _y h_z^P \end{aligned}$$

84 $$\begin{aligned} X_y^{w \mathrm {R}}= & {} \dots - q_0 \Gamma _{6a} \nabla _z h_x^P - q_0 \Gamma _{6b} \nabla _x h_z^P \end{aligned}$$

85 $$\begin{aligned} X_x^{P \mathrm {R}}= & {} \dots + q_0 \Gamma _{6a} \nabla _z m_y + q_0 \Gamma _{6b} \nabla _y m_z \end{aligned}$$

86 $$\begin{aligned} X_y^{P \mathrm {R}}= & {} \dots - q_0 \Gamma _{6a} \nabla _z m_x - q_0 \Gamma _{6b} \nabla _x m_z \end{aligned}$$

We close this subsection by pointing out that the effects described in Eqs. (81) and (82) are not restricted to polar cholesterics, but also exist in nonpolar ones. It might be easier to detect those effects there.

### Dissipative coupling terms in polar cholesterics

Inspecting the dissipation function for polar cholesterics, there is a chiral term, $R_{chir}$, which has only one gradient

87 $$\begin{aligned} R_{chir} = \Sigma ^{\mathrm {pol}}\, q_0 (s_{jik} + s_{kij}) \,m_i A_{jk}, \end{aligned}$$

which gives rise to the following contributions to the dissipative stress tensor and the quasi-current associated with the velocity difference

88 $$\begin{aligned} \sigma _{ij}^{\mathrm {D}}= & {} -\Sigma ^{\mathrm {pol}} \,q_0 (s_{ikj} + s_{jki}) \, m_k, \end{aligned}$$

89 $$\begin{aligned} X_i^{w \mathrm {D}}= & {} \Sigma ^{\mathrm {pol}} \,q_0 (s_{jik} + s_{kij}) A_{jk} \end{aligned}$$

We thus read off immediately from Eqs. (88) and (89) that applying symmetrized velocity gradients gives dissipatively rise to temporal variations of the relative velocity, while the presence of velocity differences leads to dissipative contributions to the stress tensor. Correspondingly gradients of the velocity difference will give rise to temporal variations of the density of momentum. Taken together, Eqs. (88) and (89) describe a diffusion-like behavior of $w_x$ and $w_y$ with a diffusion coefficient $q_0^2 \alpha \rho ^{-1} (\Sigma ^{\mathrm {pol}})^2$.

To obtain explicit expressions for the dissipative stresses and the quasi-current of $w_i$, we take the preferred direction, ${\hat{p}}_i$, to be parallel to the ${\hat{z}}$-direction.

Applying an external pure shear flow of the mean velocity in a plane containing the polar preferred direction, we obtain as a response temporal variations of the velocity difference

90 $$\begin{aligned} X_x^{w \mathrm {D}} =&2\, \Sigma ^{\mathrm {pol}} \,q_0 A_{zy} \end{aligned}$$

91 $$\begin{aligned} X_y^{w \mathrm {D}} =&- 2\, \Sigma ^{\mathrm {pol}} \,q_0 A_{zx} \end{aligned}$$

92 $$\begin{aligned} X_z^{w \mathrm {D}} =&0 . \end{aligned}$$

along the directions perpendicular to the shear plane.

Conversely, velocity differences lead to dissipative contributions to the stress tensor, $\sigma _{ij}^D$. Specifically we obtain the nonvanishing stresses

93 $$\begin{aligned} \sigma _{zx}^{\mathrm {D}} = \sigma _{xz}^{\mathrm {D}}= & {} - q_0 \Sigma ^{\mathrm {pol}} {\alpha w_y} \end{aligned}$$

94 $$\begin{aligned} \sigma _{zy}^{\mathrm {D}} = \sigma _{yz}^{\mathrm {D}}= & {} + q_0 \Sigma ^{\mathrm {pol}} {\alpha w_x} \end{aligned}$$

again in a helical fashion.

We emphasize that the dissipative contributions just examined are specific for two-fluid systems and have no analogue in one-component systems.

This type of dissipative cross-coupling exists also for chiral nonpolar systems.

### Reversible coupling terms in polar nematic gels

Here, we focus on reversible coupling terms in polar nematic gels between relative velocities on the one hand and elastic stresses and relative rotations on the other. These effects are specific for a two-fluid system and require a polar-preferred direction and are therefore absent in nonpolar nematic gels.

From Eqs. (59)–(62), we have for the reversible contributions of interest in this connection

95 $$\begin{aligned} X_k^{w \mathrm {R}}= & {} - \Xi _{ijk} \psi _{ij} - \Lambda \delta _{kj}^{\bot } L_j^{\bot } \end{aligned}$$

96 $$\begin{aligned} X_{ij}^{u \mathrm {R}}= & {} \Xi _{ijk} m_k \end{aligned}$$

97 $$\begin{aligned} Y_{i}^{\Omega \mathrm {R}}= & {} + \Lambda \delta _{ij}^{\bot } m_j \end{aligned}$$

with

98 $$\begin{aligned} \Xi _{ijk} = \Xi _1 {\hat{p}}_i {\hat{p}}_j {\hat{p}}_k + \Xi _2 \delta _{ij}^{\bot } {\hat{p}}_k + \Xi _3 ( \delta _{ik}^{\bot } {\hat{p}}_j + \delta _{jk}^{\bot } {\hat{p}}_i)\qquad \end{aligned}$$

where $\Xi _{ijk}$ describes the reversible connection among the relative velocity, elastic stresses and strains. Taking the preferred direction ${\hat{p}}_i$ to be parallel to the ${\hat{z}}$-direction, it is obvious that 1) $\Xi _1$ connects $w_z$, $\psi _{zz}$, and $u_{zz}$, 2) $\Xi _2$ connects $w_z$, $\psi _{xx}+\psi _{yy}$ and $u_{xx} + u_{yy}$, while 3) $\Xi _3$ connects $w_{x}$, $\psi _{xz}$ and $u_{xz}$ (or *x* replaced by *y*). These variables oscillate homogeneously with frequencies $\omega ^2 = \Xi _n^2 c_n \alpha$, with $n \in \{1,2,3\}$ for the three cases discussed above, and $c_n$ the longitudinal, the transverse, and the shear elastic modulus, respectively, and $\alpha = \rho \phi (1-\phi )$.

Of course, the dynamics of the relative velocity relaxes with rate $\xi _{ij}$, Eq. (37), leading to

99 $$\begin{aligned} \omega ^2 - i \omega \xi _n \alpha - \Xi _n^2 c_n \alpha =0 \end{aligned}$$

with $\xi _{1,2} = \xi _\parallel$ and $\xi _{3} = \xi _\perp$. Since the relaxation is expected to be strong, Eq. (99) describes an overdamped oscillation.

We close this subsection by briefly discussing the coupling of relative rotations to relative velocities $\sim \Lambda$ given in Eqs. (95) and (97). The reversible terms give rise to homogeneous oscillations with frequency $\omega ^2 = \Lambda ^2 D_1 \alpha$, with $D_1$ the stiffness coefficient for relative rotations. However, in this case not only the relative velocities are relaxing, but also the relative rotations, making this mode even more strongly overdamped.

## Summary and perspective

In this work, we have predominantly analyzed the macroscopic dynamics of polar two-fluid systems: polar nematics and gels in a nonchiral as well as in a chiral solvent. It turns out that the relative velocity as the additional dynamic variable allows for a large number of reversible and dissipative dynamic cross-coupling terms. For several of these couplings, we have outlined experimental set-ups to detect these effects, not investigated before. These include the possibility that, e.g., in polar nematics second-order temperature gradients lead to temporal variations of the relative velocity field and vice versa, gradients of the relative velocity create a heat current. In polar cholesterics, gradients of the relative velocity generate temporal changes of the polarization, and vice versa, gradients of the polarization give temporal changes of the relative velocity. In the same system, there is a dissipative coupling between the relative velocities and mean velocities involving only one gradient, i.e., mean shear flow triggers temporal changes of the relative velocity. Finally, in polar nematic gels elastic strains give rise to temporal changes of the relative velocity and, vice versa, relative velocities generate temporal changes of the strain. As a result, relative velocities can exhibit (strongly) damped oscillations.

As the systems become more complex, meaning they have typically more macroscopic variables, the number of coefficients becomes larger. This is also true as the systems go from isotropic to lower symmetries, as, for example, to uniaxial and biaxial nematics. Nevertheless, it is well known how to derive microscopic expressions for all the phenomenological parameters. In the context of nematic liquid crystals, as an example of a classical room-temperature system, this has been done by Forster [16, 17]. In a quantum-mechanical scenario, this problem was also analyzed for the superfluid phases of $^3$He [28, 54]. In both cases, the Mori–Zwanzig–Forster projector formalism was used [16, 17, 55–57]. For the equal time response (the frequency matrix), one is using classically Poisson brackets, while quantum-mechanically equal time commutators apply. In addition, the matrix of static susceptibilities and the memory matrix enter the description.

To measure the transport parameters is as a rule rather nontrivial for condensed fluid systems. While such measurements have been done for simple fluids (heat conductivity and shear viscosity) and for miscible binary mixtures (one has in addition diffusion and the Soret coefficient), the full program of measuring all dynamic transport coefficients has hardly been accomplished for any low molecular weight nematic liquid crystalline phase. All or almost all of these transport parameters have been determined for MBBA (N-(p-methoxybenzyliden)-p-butylaniline, showing the first room-temperature nematic phase) [32] and for 5CB (4-cyano-4’-pentylcyanobiphenyl), which shows a stable nematic phase at room temperature [58]. The best one can typically do practically is to study a subspace in parameter space for well-defined conditions. As an example, we mention here flow alignment in a free-standing smectic *C* film. Flow alignment of the in-plane director had been predicted theoretically [59] and has been observed subsequently [60] when applying a rotating needle to the free-standing film to apply a torque. In the same spirit, we have analyzed recently [61] flow alignment in ferromagnetic nematics. We note that frequently one can measure static susceptibilities such as, for example, Frank elastic constants in a nematic, more easily separately statically and then continue on with simple dynamic experiments.

In order to elucidate where two fluid effects can also become important in addition for polar nematics and polar cholesterics, it is useful to remind the reader in which cases solvent effects, etc., have become important for the description of usual nematics and cholesterics. For ordinary nematics, an outstanding flow problem for several decades has been the breakdown of flow alignment for nematic phases having smectic clusters regardless whether a smectic phase follows at lower temperatures or not. Quite recently, it has been shown [13] that a two fluid description including smectic clusters can naturally account for this phenomenon. Along the same lines, it has been pointed out that clusters of various types can account for spatial heterogeneities as the glass transition is approached from above [14]. Naturally this will also be the case above the glass transition in liquid crystalline polymers.

There has been another long-standing puzzling feature in the electric domain of nematics with smectic clusters, namely a sign change in the anisotropy for the electric conductivity [62–65]. This also lead in turn to electroconvective patterns unknown from other systems [66]. In Ref. [13], it has been demonstrated how a two-fluid picture can account for the change in the anisotropy of the electric conductivity, which had no other explanation before.

The electric effects of ionic impurities in classical thermotropic nematics also have a long prehistory, in particular in the field of electroconvection. To account for these effects, the group of Kramer generated WEM (Weak Electrolyte Model) [67, 68]. It turns out that ionic impurity concentrations and their transport also play an important role in the explanation of experimental results [68, 69].

When it comes to polar nematics and polar cholesterics, the number of well-controlled *dynamic* experiments on the influence of ions and their motion as well as of clusters appears to be almost zero. Surely colloidal rods in water might not be the first choice to reveal two fluid effects, but they are a rather special case of classical nematics with a solvent. We rather think of nematic polymeric and elastomeric systems and their chiral analogues as they are abundant in biological systems.

Clearly Ref. [29] is not sufficient to keep track of any of the effects discussed in the present paper, and it therefore appears to be essential to test the predictions made here. Surely for ferroelectric nematic systems as they have been studied recently, electric effects can be expected to be important and relevant, simply because they cannot be possibly spatially homogeneous in the bulk as already pointed out by the present authors more than thirty years ago [35].

A topic we leave for future investigations is the impact of two fluid effects on permeation flows [32, 70, 71] and their generalizations [72]. In the simplest case, namely for the flow with approximately constant velocity through a fixed cholesteric structure [32, 70], one obtains a plug flow with strongly enhanced apparent viscosity [71]. It will be most interesting to see how these effects are modified in the presence of two fluid effects for the plug flow [32, 70] and it generalizations [72].

In this manuscript, we have dealt with the 2-fluid behavior of systems with preferred directions that break inversion symmetry, but are time reversible. Systems of interest are mainly coming from liquid crystal physics, but also from biological applications. It is natural to also look at the two-fluid macroscopic dynamic behavior of preferred directions that are inversion symmetric, but break time reversal symmetry, as is found in magnetically ordered systems. And this class of systems already exists experimentally, namely ferromagnetic nematics [73–77] and ferromagnetic cholesterics [78–80]. Both classes of systems can be viewed as suspensions of magnetic platelets in a nematic or a cholesteric liquid crystal as a solvent. So far the focus experimentally and theoretically has been on the macroscopic dynamics of one-component ferromagnetic nematics and ferromagnetic cholesterics [36, 61, 81–83] generalizing earlier work on the macroscopic dynamics of ferronematics [84, 85]. As for the two-fluid aspects, there appears to be no work on the dynamics of ferromagnetic nematics and ferromagnetic cholesterics, while some static experimental aspects of this type of behavior including converse magneto-electric effects and magneto-optic effects have been already examined for ferromagnetic nematics in the literature [74]. Only rather recently investigations of the macroscopic dynamic aspects of magnetic two-fluid systems have been started for magneto-rheological fluids [86].

Another class of two-fluid systems of interest that should be investigated in a next step are two-fluid systems with anisotropic clusters. So far the effect of clusters on macroscopic dynamics has been studied exclusively for isotropic clusters close to a transition such as above the glass transition or in the vicinity of a second-order phase transition [14] as well as for clusters in a nematic phase above the smectic–nematic phase transition [13].
